# Estimation of flavoniods, antimicrobial, antitumor and anticancer activity of *Carissa opaca* fruits

**DOI:** 10.1186/1472-6882-13-372

**Published:** 2013-12-27

**Authors:** Sumaira Sahreen, Muhammad Rashid Khan, Rahmat Ali Khan, Naseer Ali Shah

**Affiliations:** 1 Botanical Sciences Division, Pakistan Museum of Natural History, Garden Avenue, Shakarparian, Islamabad, Pakistan; 2 Department of Biochemistry, Faculty of Biological Sciences, Quaid-i-Azam University Islamabad, Islamabad, Pakistan; 3 Department of Biotechnology, Faculty of Biological Sciences, University of Science and Technology, 28100 Bannu, KPK, Pakistan

**Keywords:** *Carissa opaca*, High performance liquid chromatography, *Escherichia coli*, Anticancer

## Abstract

**Background:**

*Carissa opaca* Stapf ex Hanes fruits is traditionally used in the treatment of asthma, hepatitis and microbial infections. The present study was arranged to investigate the antimicrobial, cytotoxic and antitumor activity of various fractions of *C. opaca* extract and its bioactive metabolites responsible for that activity.

**Methods:**

To characterize various fractions of *C.opaca* antibacterial, antifungal, cytotoxic and antitumor assays are used. Eight strains of bacteria including *Bacillus subtilis*, *Enterobactor aerogenes*, *Escherichia coli*, *Klebsiella pneumoniae*, *Micrococcus luteus*, *Pseudomonas aeroginosa*, *Salmonella typhy*, and *Staphylococcus aureus* and four strains of fungal viz: *Aspergillus flavus*, *Aspergillus fumigatus, Aspergillus niger* a*nd Fusarium solani* are used. Brine shrimps and potato dics are used for anticancer and antitumor potency of extract. High performance liquid chromatography (HPLC) is utilized for determination of bioactive metabolites responsible for the activity.

**Results:**

HPLC chromatogram revealed the presence of orientin, isoquercetin, myricetin and apigenin. Various fractions of *C. oapca* showed significant antibacterial, antitumor and anticancer activity. In case of *C. opaca* fruit inhibition growth of *Aspergillus niger* was ranged between 23.2 ± 1.36% to 43.3 ± 2.39%, *Aspergillus flavus* ranged between 27.6 ± 1.39% to 65.6 ± 3.44%, *Aspergillus fumigatus* ranged between 13.2 ± 1.00% to 52.4 ± 1.54% and *Fusarium solani* ranged between 10.5 ± 1.02% to 14.6 ± 1.74%.

**Conclusion:**

It can be concluded that, various fractions of *C.opaca* are accessible source of ethno pharmacy as they are consumed in different areas of Pakistan with ultimate health compensations.

## Background

*Carissa opaca* Stapf ex Hanes is widely distributed from Punjab-Himalayas up to 6000 ft, in Murree (Pakistan) as well as in some areas of India, Burma and Sri Lanka [1] traditionally used in the treatment of asthma [2], cardiac dysfunction [3], microbial infection [4], cough, diarrhea and fever [5]. In spite of its popular uses, there is no information about secondary metabolites. The scientific testimonials about discovery of plants as reservoirs of bioactive substances for the deterrence of degenerative and chronic ailments are in frequent progress. Indeed, the origin of various remedial therapies is due to plant secondary metabolites. Plants that possess flavonoids have strong anti-inflammatory, antiviral, antioxidant, antiallergenic, anti-fungal, antibacterial, anticancer, cytotoxic and hepatoprotective activities thus, generated curiosity about flavonoid containing plants [6,7]. In the recent years, medicinal plants are supplementary focused regarding to therapeutic properties of traditional medicines in the treatment of various incoming diseases. It has become necessary to find out new antimicrobial drugs having diverse chemical structures so that they can deactivate new lethal microorganisms [8,9]. Hence, an outstanding chemical diversity found in botanicals may be of great potential to relate with new antimicrobial agents. These antimicrobial agents are functional against plant and human pathogens. Previous literature reports that the bioassay-guided fractionation and antimicrobial activity of plant extracts yield active principles [10]. Antimicrobial agents of botanicals with massive remedial potential to heal many infectious diseases are void of side effects in comparison to synthetic drugs. Medicinal plants offer a considerable novel antimicrobial therapeutic potential and adjunct treatment against resistant microbial strains. Hence, it has become crucial to explore new antimicrobial agents from natural resources. Although the ethnobotanical literature about *C. opaca* is well acknowledged, yet very little scientific information about its activity against microorganism is available. Cancer, being a world-wide health problem, is treated with clinical therapies including chemotherapy, surgery, radiation therapy and so on by using synthetic medicines [11]. Toxic side effects of synthetic medicines have made a comeback for herbal medicine to improve our present and future health needs. Thus, development of simple suitable and economical assay systems for prescreening of a wide range of samples can seek advantageous as alternatives to prolonged animal trials for the discovery of new anticancer drugs. Various bioassay methods have been assessed for the antitumor or anticancer activity of plant extract/fractions or compounds. Several famous and noteworthy discoveries like vincristine, vinblastine the podophyllotoxin derivatives and 10-hydroxy-campothecin are the outcome of these methods and also derived from botanicals. Sharma [11] reported that diterpene resins of Croton species have been possibly prove to be useful in cancer therapy. It is reported that, there is a strong correlation between human nasopharyngeal carcinoma and brine shrimp toxicity. Brine shrimp lethality assay (BSLA) provide a front-line screen to back up more specific and expensive bioassays for isolation of active compounds due to inexpensive and rapid means of standardization of bioactivity in heterogeneous botanical products [12]. Brine shrimp lethality test is going to be practiced routinely as an indicator of possible cytotoxic properties in order to reveal new anticancer drugs. Cytotoxic assays are being frequently applied for the analysis of mycotoxins, anesthetics, dinoflagelate toxins and marine toxicants. Thus, using these cytotoxic assays, many novel pesticidal and anticancer natural products have been documented [13,14]. Antitumor potato disc assay using *Agrobacterium tumefaciens* is considered as quick, low-priced and consistent prescreening for antitumor agents of botanicals [15]. It is anticipated that this study would lead to formulation of new and more potent antimicrobial and anticancer drugs of natural origin.

## Methods

### Plant collection

Fruits of *C. opaca* were obtained in March-April 2011 from Abbottabad of Northern Pakistan, recognized by their local names and validated by Dr. Mir Ajab Khan, Department of Plant Sciences, Quaid-i-Azam University, Islamabad, Pakistan. A voucher specimen with Accession No. 24561 (C. opaca) was deposited at the Herbarium of Pakistan.

### Plant extraction

The collected plant samples were cleaned to get rid of dust particles and then dried under shade for one to two weeks and made into fine powder. 2 kg of sample was extracted twice with 5 L of 95% methanol at 25°C for 48 h. For filtration Whatman No. 1 filter paper was used and then filtrate was concentrated on rotary evaporator (Panchun Scientific Co., Kaohsiung, Taiwan) under reduced pressure at 40°C. In order to resolve the compounds with escalating polarity, a part of the extract was suspended in distilled water and subjected to liquid-liquid partition by using solvents in a sequence of n-hexane, ethyl acetate, chloroform, butanol and water to obtain their soluble fractions. After fractioning, the solvent of respective fractions was also evaporated by rotary evaporator Extract was dried and then stored at 4°C for further *in vitro* investigation.

### High performance liquid chromatogrhy (HPLC) of methanolic fraction

#### Sample preparation

50 mg of *C. opaca* powder was extracted with 6 ml of 25% hydrochloric acid and 20 ml methanol for 1 hr. The obtained extract was filtered to a volumetric flask. The residue was heated twice with 20 ml of methanol for 20 min to obtain the extract. The combined extract was diluted with methanol to 100 ml. 5 ml portion of the solution was filtered and then was transferred to a volumetric flask and diluted with 10 ml of methanol. The sample (10 μl) was injected into the HPLC apparatus.

#### HPLC determination

Samples were analyzed on Agilent HPLC system. Separation was carried out through column 20RBAX ECLIPSE, XDB-C18, (5 μm; 4.6 × 150 mm, Agilent USA) with UV-VIS Spectra-Focus detector, injector-auto sampler. Solvent A (0.05% trifluoroacetic acid) and solvent B (0.038% trifluoroacetic acid in 83% acetonitrile (v/v) with the following gradient: 0-5 min, 15% B in A, 5-10 min, 70% B in A, 10-15 min, 70% B in A are used for separation The flow rate was 1 ml/min and injection volume was 10 μl. Eleven standard compounds including rutin, myricetin, vitexin, orientin, hyperoside, isovitexin, isoquercetin, luteolin, apigenin, kaempherol, and luteolin-7-glucoside were run for comparative detection and optimized. The calibration curves were defined for each compound in the range of sample quantity 0.02-0.5 μg. All samples were assayed in triplicate.

### Antibacterial assay

The antibacterial potency of various fractions of *C. opaca* fruits was carried out through protocol of (Bagamboula *et al*. [16] using eight strains of bacteria were used; which were *Bacillus subtilis* (ATCC 6633), *Enterobactor aerogenes* (ATCC 13048), *Escherichia coli* (ATCC 15224), *Klebsiella pneumoniae* (MTCC 618), *Micrococcus luteus* (ATCC 10240), *Pseudomonas aeroginosa* (ATCC 27853), *Salmonella typhy* (ATCC 0650), and *Staphylococcus aureus* (ATCC 6538). Antibacterial activities were calculated as a mean of 3 replicates. Diameter of the clear zones, showing no bacterial growth, around each well was measured with the help of vernier caliper. Triplicate plates were prepared for each sample.

### Antifungal assay

The agar tube dilution method was used for antifungal activity of plant extracts was determined according to the protocol reported by Duraipandiyan and Ignacimuthu [17] using *Aspergillus flavus (0064)*, *Aspergillus fumigatus (66), Aspergillus niger (0198)* a*nd Fusarium solani (0300). *Tubes were prepared in triplicate for each fungus species. Percentage inhibition of fungal growth for each concentration of fractions was determined by the following formula;

$$\begin{array}{l} \mathrm{Percentage}\;\mathrm{inhibition}\;\mathrm{of}\;\mathrm{fungal}\;\mathrm{growth};\\ =(100-\mathrm{linear}\;\mathrm{growth}\;\mathrm{in}\;\mathrm{test}\;\left(\mathrm{mm}\right)/\\ \;\mathrm{linear}\;\mathrm{growth}\;\mathrm{in}\;\mathrm{control}\;\left(\mathrm{mm}\right)\times 100 \end{array}$$

### Cytotoxic brine shrimp assay

Various fractions of *C.opaca* were characterized according to Meyer-Alber *et al.*[18] using brine shrimps hatched in saline. 10-1000 PPM was incubated with brine shrimps. After 24 h of incubation survivors were counted with help of 3x magnifying glass and calculation was done using Abbot^’^s formula;

$$\begin{array}{l} \%\;{\mathrm{Death}}_{=}\left(\mathrm{Sample}-\mathrm{control}/\mathrm{control}\right)\times 100\\ {\mathrm{LD}}_{50}\;\mathrm{was}\;\mathrm{determined}\;\mathrm{through}\;\mathrm{prism}\;\mathrm{graph}\;\mathrm{pad}\;\mathrm{software}. \end{array}$$

### Antitumor potato disc assay

The potato disc method was used for anti-tumor activity of plant extracts as reported by Ferrigini *et al.*[19]. Number of tumors per disc was counted and percentage inhibition for each concentration was determined as follows:

$$\begin{array}{l} \%\mathrm{age}\;\mathrm{inhibition}=100-[\left(\mathrm{average}\;\mathrm{number}\;\mathrm{of}\;\mathrm{tumors}\;\mathrm{of}\;\mathrm{sample}\right)/\\ \;\left(\mathrm{Average}\;\mathrm{number}\;\mathrm{of}\;\mathrm{tumors}\;\mathrm{of}–\mathrm{ve}\;\mathrm{control}\right)]\times 100 \end{array}$$

IC_50_ was recorded using graph prism pad software.

### Statistical analysis

Data of *in vitro* assays recorded were analyzed with help computerized Graph prism pad software to determined EC_50_, IC_50_ and LD_50_.

## Results

### High performance liquid chromatography (HPLC) of methanolic fractions of *C.opaca*

**Figure 1:**
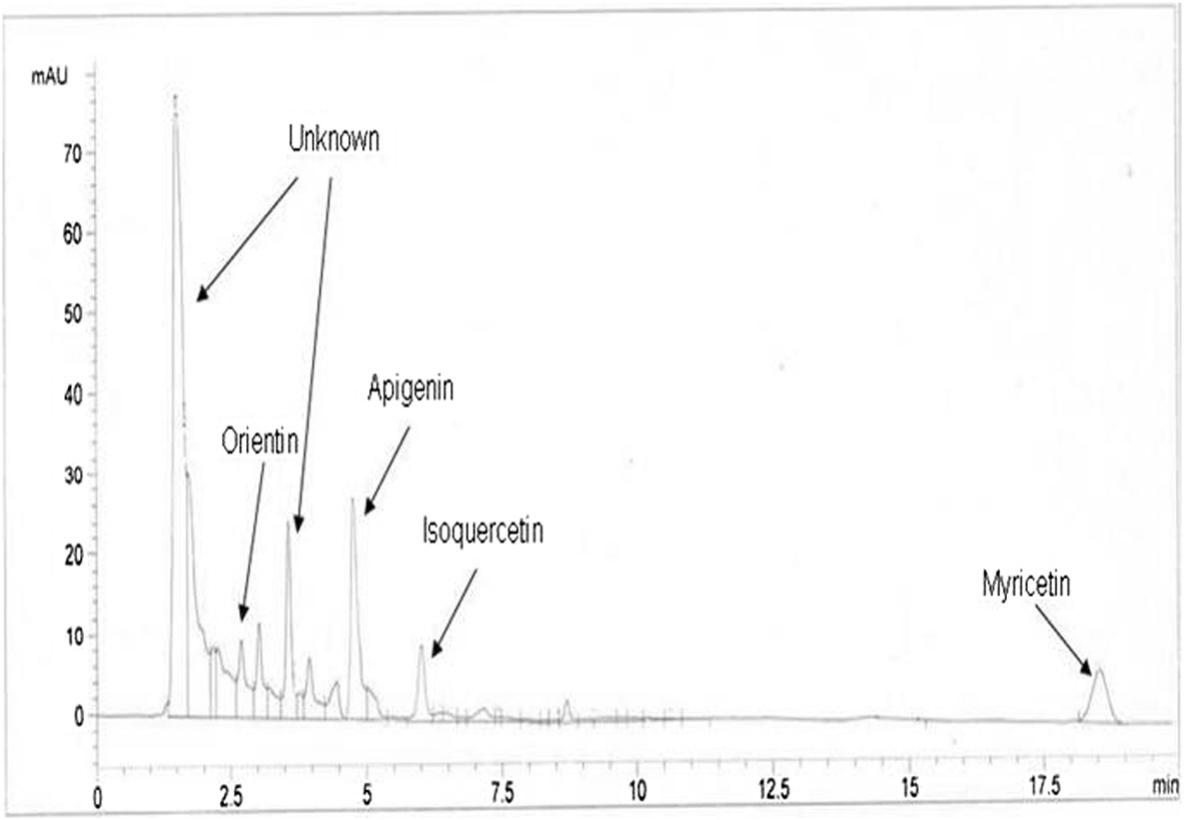
**HPLC flavonoid profile of methanolic fraction of *C. opaca *fruits.** Conditions: mobile phase, ACN-dH_2_O; flow rate, 1 ml min^-1^; detection wave length, 220 nm; column temperature, 36°C; injection volume, 10 μl.

In the present study, HPLC-UV was preferred for the qualitative as well as quantitative analysis of methanolic fraction of *C. opaca* fruit. First of all, the experimental conditions were optimized to get the chromatograms with better resolution within a short resolution time and maximum UV absorption of sample. Hence, flavonoid standard compounds and the plant samples were quantified by assimilation of peak areas at 220 nm within runtime of 20 minutes. The standard solutions were injected in duplicate and the curves were constructed with the averages showing good linear correlation coefficient in the concentration range. All the standard flavonoids confirmed good linearity in a bit large concentration range. The conditions used directed towards the good separation of peaks that may be identified in the chromatogram as apigenin (R_t_=4.7), myricetin (R_t_=18.5), vitexin (R_t_=2.5), orientin (R_t_=2.75), hyperoside (R_t_=12.5), isovitexin (R_t_=3.7), isoquercetin (R_t_=6), rutin (R_t_=8.7), luteolin (R_t_=2.01), kaempherol (R_t_=3.4), luteolin-7-glucoside (R_t_=1.6) (Table 1). A sample of 10 μl of solution was injected to the instrument. Identification was done by comparing the obtained peaks of chromatogram of samples with the peaks of standard flavonoids in respect to retention time and UV-spectra. The chromatogram determining flavonoids components of methanolic fraction of *C. opaca* fruit in Figure 1. Table 2 summarized the flavonoids found in the methanolic fraction of *C. opaca* fruits as well as their retention time. Data indicated that methanolic fraction showed the presence of orientin, isoquercetin, myricetin and apigenin. There were some peaks having different retention time could not be identified; however, based on their chromatographic behaviors and UV spectra, they may correspond to unknown flavonoids compounds as presented in respective chromatogram.

**Table 1:** Linear regression analysis of eleven standard flavonoids

**Compound**	**Retention time**	**Regression analysis**	**R**	**Linear range (ppm)**	**LOD (ppm)**
Rutin	8.7	y = 12571.3x-16.62	0.9873	10-250	3
Myricetin	18.5	y = 9643.4x-11.07	0.9919	10-200	3.5
Vitexin	2.5	y = 23085.1x + 3.72	0.9932	10-100	1
Orientin	2.75	y = 36421.0x + 2.88	0.9869	25-500	3.2
Hyperoside	12.5	y = 22758.9x + 1.56	0.9865	10-250	2.3
Isovitexin	3.7	y = 31604.2x + 0.98	0.9741	5-150	1.8
Luteolin	2.01	y = 19348.6x + 2.08	0.9532	5-100	2.1
Isoquercetin	6	y = 26785.6x + 1.60	0.9616	5-500	2.5
Apigenin	4.7	y = 10623.5x-9.82	0.9765	25-250	1.2
Kaempherol	3.4	y = 26182.8x- + 2.33	0.9417	10-500	1.34
Luteolin-7-glucoside	1.6	y = 11434.6x-10.72	0.9536	5-100	0.68

Mean ± SE (n = 3).

**Table 2:** **HPLC flavonoids profile of methanolic fraction of *C. opaca *** **fruits**

**Compound**	**Retention time**	**Concentration μg/mg of dry weight**
Orientin	2.75	0.387
Isoquercetin	6	0.118
Myricetin	18.5	0.069
Apigenin	4.7	0.188

Mean ± SE (n = 3).

### Antibacterial screening (MIC)

The *in vitro* antibacterial activity of various fractions of *C. opaca* was evaluated by minimum inhibitory concentration (MIC) values. The MIC is the lowest concentration of test sample to inhibit visible growth of bacteria. The antibacterial activity was tested against both gram-negative bacteria as well as gram-positive bacteria. Table 3 describes the antibacterial activity as MIC value of various fractions of plant samples against tested bacteria. The results obtained from the present study for all tested bacteria ranged from 0.1 to 10 mg/ml. Gram-positive bacteria such as *Staphylococcus aureus* was inhibited by MIC value (1 mg/ml) of HFC, MFC, 2.5 mg/ml of CFC, 5 mg/ml of BFC, while rest of the fractions didn’t inhibit the growth of *Staphylococcus aureus*. On the other hand, *Bacillus subtilis* was inhibited by HFC, (0.1 mg/ml); CFC, MFC (1 mg/ml); EFC (1.2 mg/ml); AFC, (2.5 mg/ml) while remaining fractions did not inhibit the growth of the respective bacteria. In case of gram negative bacteria *Klebsiella pneumoniae*’s growth was inhibited by EFC, CFC, MFC, AFC (1 mg/ml) while remaining fractions did not inhibit the growth of the respective bacteria. *Pseudomonas aeroginosa* was inhibited by HFC (1 mg/ml); CFC, MFC, AFC (10 mg/ml) and rest of the fractions inhibited the growth at 0.1 mg/ml. Growth of *Salmonella typhy* was inhibited by CFC, AFC (0.5 mg/ml) while rest of the fractions inhibited the growth at 0.1 mg/ml. However, HFC, EFC and BFC inhibited the growth of *Enterobacter aerogenes* with MIC (1 mg/ml); CFC (0.1 mg/ml); MFC (5 mg/ml) while remaining fractions did not show inhibition of the concerned bacteria. MIC against *Micrococcus luteus* and *Escherichia coli* had no value, showing no antibacterial activity of any of the fractions.

**Table 3:** **Antibacterial screening as MIC (mg/ml) of various fractions of *C. opaca *** **against pathogenic bacterial strains**

**Extracts**	***B. subtilis***	***E. aerogenes***	***E. coli***	***K. pneumoniae***	***M. luteus***	***S. typhy***	***P. aeroginosa***	***S. aureus***
MFC	1	5	-	1	-	0.1	10	1
HFC	0.1	1	-	0.1	-	0.1	1	1
EFC	1.2	1	-	1	-	0.1	0.1	-
CFC	1	0.1	-	1	-	0.5	10	2.5
BFC	-	1	-	0.1	-	0.1	0.1	5
AFC	2.5	1	-	1	-	0.5	10	10

Mean ± SE (n = 3).

### Antifungal screening

The *in vitro* antifungal activity of various fractions of *C. opaca* fruit was evaluated against four fungal strains as shown in Table 4. In case of *C. opaca* fruit inhibition in growth of *A. niger* was ranged between 23.2 ± 1.36% to 43.3 ± 2.39%, *A. flavus* ranged between 27.6 ± 1.39% to 65.6 ± 3.44%, *A. fumigatus* ranged between 13.2 ± 1.00% to 52.4 ± 1.54% and *F. solani* ranged between 10.5 ± 1.02% to 14.6 ± 1.74%.

**Table 4:** **Antifungal screening of various fractions of *C. opaca *** **fruit against pathogenic fungal strains**

**Extracts**	**% inhibition of various fungal species**			
	***A. niger***	***F. solani***	***A. flavus***	***A. fumigatus***
HFC	23.2 ± 1.36	13.4 ± 2.35	34.7 ± 2.50	13.2 ± 1.00
EFC	40.7 ± 2.00	10.6 ± 1.43	29.8 ± 1.62	20.4 ± 1.37
CFC	26.5 ± 2.66	14.6 ± 1.98	29.9 ± 2.18	38.1 ± 1.88
BFC	32.4 ± 1.08	10.5 ± 1.02	27.6 ± 1.39	47.0 ± 1.72
MFC	43.3 ± 2.39	14.6 ± 1.74	53.2 ± 1.42	44.6 ± 1.43
AFC	41.2 ± 1.54	13.8 ± 1.42	65.6 ± 3.44	52.4 ± 1.54

Mean ± SE (n = 3).

### Cytotoxic screening

Cytotoxic screening using brine shrimps bioassays was carried out to provide important preliminary data to select plant extracts. Cytotoxic effect of various fractions of *C. opaca* fruit was evaluated against brine shrimps growth under controlled condition as presented in Table 5. Generally this assay indicated that the survival of brine shrimps was inversely proportional to the concentration of different fractions. LD_50_ was the lowest concentration of test sample at which 50% death of shrimps occurred. Data of present study indicated that the order of LD_50_ of various fractions of *C. opaca* fruits was CFC > MFC > AFC > BFC > EFC > HFC.

**Table 5:** **Cytotoxic screening of various fractions of *C. opaca *** **fruit against brine shrimps after 24 hours**

**Extracts**	**10 ppm**	**100 ppm**	**1000 ppm**	**LD**_**50 **_**(ppm)**
HFC	29.37 ± 1.23	45.28 ± 1.33	60.38 ± 1.43	420
EFC	34.62 ± 1.11	50.45 ± 1.01	62.44 ± 1.89	100
CFC	48.21 ± 2.11	80.42 ± 2.41	88.44 ± 2.11	20
BFC	29.34 ± 3.42	57.3 ± 2.98	72.21 ± 2.33	80
MFC	32.13 ± 2.45	71.45 ± 3.42	89.43 ± 1.23	55
AFC	40.11 ± 2.99	56.34 ± 2.19	67.42 ± 2.66	60

Mean ± SE (n = 3).

### Antitumor screening

Potato disc assay was performed to reveal the antitumor potential of various fractions of *C.* opaca fruit. This assay has been routinely used due to its rapid, economical and statistically valid prescreening for antitumor activity. In the present study, *Agrobacterium tumefaciens* (AT 10) was used against three different concentrations (10, 100 and 1000 ppm) of different fractions of *C.* opaca fruit. Tumor inhibition was calculated through IC_50_ value. The IC_50_ values of antitumor potential of plant samples are summarized in Table 6. Data obtained indicated that the order of IC_50_ of various fractions of *C. opaca* fruits for antitumor screening was BFC > AFC > CFC > MFC > EFC and HFC.

**Table 6:** **Antitumor screening of various fractions of *C. opaca *** **fruit against potato disc tumor after 21 days**

**Extracts**	**10 ppm**	**100 ppm**	**1000 ppm**	**IC**_**50 **_**(ppm)**
HFC	12.76 ± 1.78	23.22 ± 1.00	30.98 ± 1.33	>1000
EFC	19.11 ± 1.62	27.44 ± 1.02	39.56 ± 1.98	>1000
CFC	25.76 ± 2.19	69.35 ± 2.36	83.55 ± 3.15	60
BFC	40.56 ± 1.82	65.17 ± 2.33	72.44 ± 2.32	40
MFC	32.61 ± 1.26	60.45 ± 1.28	80.33 ± 2.99	65
AFC	28.27 ± 2.41	70.54 ± 2.52	76.00 ± 2.54	55
Positive control	49.2 ± 1.2	69.5 ± 2.3	88.9 ± 3.2	11

Mean ± SE (n = 3).

## Discussion

Phytochemicals screening is carried out to allow isolation of novel or valuable components with potential activities at the earliest stages thus economically very important. Present study exposed the naturally occurring dietary substances such as alkaloids, flavonoids, saponins, tannins and other bioactive metabolites in various fractions of plant samples. Methanol fraction of *C.apaca* showed the presence of important flavonoid i.e., orientin, isoquercetin, myricetin and apigenin. possess anti-disease wealth particularly minimizes the risk of cancer [20]. In literature, many medicinal plants indicated their strength through antimicrobial and anticancer behavior that was endorsed with high concentration of flavonoids and alkaloids [21,22]. Flavonoids, a large group of phenolics also described as nature loving drugs, owned various biological actions [23] like anti-inflammatory aptitude flavonoid containing Chinese medicine [24,25]. Thereby, presence of flavonoids in *C. opaca* can verify their folkloric use against cancer, skin infections and rheumatism. This study is a preliminary estimation of antimicrobial activity of *C. opaca* fruits. The results of the antibacterial and antifungal activity vary with the fractions of the plant studied. It could result in the discovery of new chemical classes that may possibly proceeded as biochemical tools to study infectious diseases and endowed with selective agents for antibiotics. Our results suggest that antimicrobial activity of plant fractions doesn’t depends on only phenolics but other secondary metabolites are also involved. Previous studies revealed that plant extracts have bactericidal effects and consequently seems to be promising in for treatment of microorganisms [26]. Another interesting point is that none of the fractions showed activity against the Gram positive bacteria, *Micrococcus luteus* and the Gram negative bacteria, *Escherichia coli* and consequently for rest of the strains they were active. Flavonoids are known to possess antibacterial activity and, according to Tsuchiya *et al*. [27], dihydroxylation of the A and B rings and substitution with a specific aliphatic group are important for the antimicrobial activity of certain flavanones. Our results of antibacterial activity are in accord to the results of Kil *et al*. [28] and Martini and Eloff [29] who reported antimicrobial activity of different fractions of *Sorghum* and *Cicer erythrophyllum*, respectively. As previous reports suggest that Gram negative bacteria are additionally resistant as compare to Gram positive bacteria, the non polar fractions were the only ones to show activity against Gram-negative bacteria [30]. But in our findings other polar fractions also showed higher activity against Gram-negative bacteria representing combined effects of different phytochemicals found in each fraction. It may be possible that polarity of the polar chemicals like tannins, flavonoids and alkaloids present in fractions can interact with the chemical composition of the polar cell structure of this type of bacteria. Flavonoids as well as triterpenoids and alkaloids could be responsible for the antimicrobial activities [31]. For the investigation of anticancer bioactive compounds, biological assays especially brine shrimp lethality assay (BSLA) is considered as necessary and suitable tools. The variation in BSLA results showed the presence and synergistic effects of a variety of bioactive compounds. Our observations were in accordance with Peteros and Uy [23] who reported that the quantity and quality of secondary metabolites like flavonoids, tannins etc. determine the potential of cytotoxicity of extracts, possibly responsible for difference in results. According to Meyer *et al.*[18] who categorized crude extracts and pure compounds into toxic (LC_50_ value < 1000 ppm) and non-toxic (LC_50_ value > 1000 ppm), all tested showed good brine shrimp larvicidal activity. Moreover, Peteros and Uy [23] signified the presence of effective cytotoxic substances of plant extracts having LC_50_ values < 100 ppm to brine shrimp lethality.

## Conclusion

Most of the tested fraction showed substantial antimicrobial, antitumor and anticancer activities, which is in accordance with the spacious use of tested plant samples in primary health care. It can be concluded that, studied plant parts are accessible source of ethno pharmacy as they are consumed in different areas of Pakistan with ultimate health compensations.

## Competing interests

The authors declare that they have no competing interests.

## Authors’ contributions

SS made significant contribution to acquisition of data, analysis, drafting of the manuscript. MRK has made substantial contribution to conception and design, interpretation of data, drafting and revising the manuscript for intellectual content. RAK (ORCID ID: 0000-0003-0453-2090) participated in the design and collection of data and analysis. All authors read and approved the final manuscript.

## Pre-publication history

The pre-publication history for this paper can be accessed here:

http://www.biomedcentral.com/1472-6882/13/372/prepub
